# 2,5-Dihydroxy­benzaldehyde 4-methyl­thio­semicarbazone

**DOI:** 10.1107/S1600536808018801

**Published:** 2008-06-28

**Authors:** Kong Wai Tan, Chew Hee Ng, Mohd Jamil Maah, Seik Weng Ng

**Affiliations:** a Department of Chemistry, University of Malaya, 50603 Kuala Lumpur, Malaysia; b Faculty of Engineering and Science, Universiti Tunku Abdul Rahman, 53300 Kuala Lumpur, Malaysia; c Department of Chemistry, University of Malaya, 50603 Kuala Lumpur, Malaysia

## Abstract

The planar mol­ecules of the title compound, C_9_H_11_N_3_O_2_S, are linked into a supra­molecualr chain *via* O—H⋯S hydrogen bonds. These chains are connected into a two-dimensional array via N—H⋯O hydrogen bonds; an intra­molecular O—H⋯N hydrogen bond is also present.

## Related literature

For the medicinal activity of 2,5-dihydroxy­benzaldehyde thio­semicarbazone, see: Libermann *et al.* (1953 ▶); Taniyama & Tanaka (1965 ▶); Xue *et al.* (2007 ▶). For the structure of 2-hydroxy­benzaldehyde 4-methyl­thio­semicarbazone, see: Vrdoljak *et al.* (2005 ▶). For the structure of 3,4-dihydroxy­benzaldehyde 4-ethyl­thio­semicarbazone, see: Kayed *et al.* (2008 ▶).
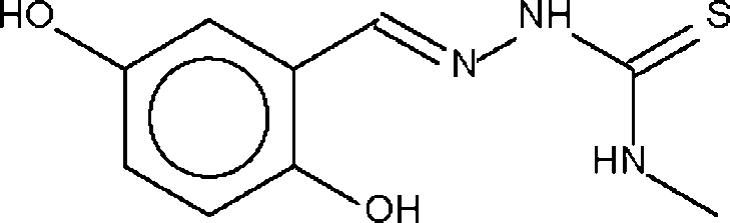


## Experimental

### 

#### Crystal data

C_9_H_11_N_3_O_2_S
*M* *_r_* = 225.27
Triclinic, 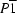

*a* = 5.9932 (4) Å
*b* = 8.5207 (6) Å
*c* = 10.3272 (6) Å
α = 78.552 (4)°
β = 74.181 (4)°
γ = 81.743 (4)°
*V* = 495.06 (6) Å^3^
*Z* = 2
Mo *K*α radiation
μ = 0.31 mm^−1^
*T* = 100 (2) K
0.24 × 0.16 × 0.02 mm

#### Data collection

Bruker SMART APEX diffractometer
Absorption correction: multi-scan (*SADABS*; Sheldrick, 1996 ▶) *T*_min_ = 0.929, *T*_max_ = 0.994
4189 measured reflections
2258 independent reflections
1580 reflections with *I* > 2σ(*I*)
*R*_int_ = 0.050

#### Refinement

*R*[*F* ^2^ > 2σ(*F* ^2^)] = 0.054
*wR*(*F* ^2^) = 0.123
*S* = 1.03
2258 reflections
153 parameters
4 restraints
H atoms treated by a mixture of independent and constrained refinement
Δρ_max_ = 0.32 e Å^−3^
Δρ_min_ = −0.47 e Å^−3^

### 

Data collection: *APEX2* (Bruker, 2007 ▶); cell refinement: *SAINT* (Bruker, 2007 ▶); data reduction: *SAINT*; program(s) used to solve structure: *SHELXS97* (Sheldrick, 2008 ▶); program(s) used to refine structure: *SHELXL97* (Sheldrick, 2008 ▶); molecular graphics: *X-SEED* (Barbour, 2001 ▶); software used to prepare material for publication: *publCIF* (Westrip, 2008 ▶).

## Supplementary Material

Crystal structure: contains datablocks I, global. DOI: 10.1107/S1600536808018801/tk2278sup1.cif

Structure factors: contains datablocks I. DOI: 10.1107/S1600536808018801/tk2278Isup2.hkl

Additional supplementary materials:  crystallographic information; 3D view; checkCIF report

## Figures and Tables

**Table 1:** Hydrogen-bond geometry (Å, °)

*D*—H⋯*A*	*D*—H	H⋯*A*	*D*⋯*A*	*D*—H⋯*A*
O1—H1*o*⋯N3	0.84 (3)	1.97 (2)	2.698 (3)	144 (3)
O2—H2*o*⋯S1^i^	0.84 (3)	2.46 (2)	3.182 (2)	144 (3)
N2—H2*n*⋯O1^ii^	0.84 (3)	2.47 (3)	3.111 (3)	134 (3)

Symmetry codes: (i) 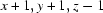; (ii) 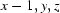.

**Fig. 1.**
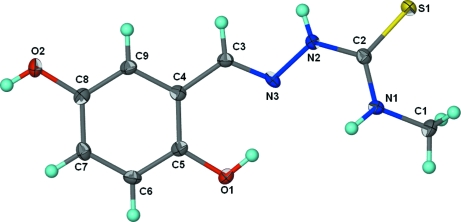
Thermal ellipsoid plot of (I) at the 70% probability level showing atom labeling. Hydrogen atoms are drawn as spheres of arbitrary radii.

## supplementary crystallographic information

### Comment

The title compound (I, Fig. 1) possesses useful medicinal properties (Libermann *et al.*, 1953; Taniyama & Tanaka, 1965; Xue *et al.*, 2007). The molecules are linked into supramolecular chains by O-H···S hydrogen bonds involving the O2-hydroxy group, Table 1. The hydrogen-bonded chains are consolidated into a layer motif via N-NH···O hydrogen bond, involving the O1-hydroxy group. An intramolecular N-H···O hydrogen bond, also involving the O1-hydroxy group is also noted. In contrast, 2-hydroxybenzaldehyde 4-methylthiosemicarbazone, which features an intramolecular O–H···N hydrogen bond, adopts a chain structure (Vrdoljak *et al.*, 2005) as it lacks a second hydroxy substituent for layer formation.

### Experimental

4-Methylthiosemicarbazide (0.11 g, 1 mmol) and 2,5-dihydroxybenzaldehyde (0.14 g, 1 mmol) were heated in ethanol (10 ml) for 1 h. Slow evaporation of the solvent yielded yellow crystals of (I).

### Refinement

Carbon-bound H-atoms were placed in calculated positions (C—H 0.95 to 0.98 Å) and were included in the refinement in the riding model approximation, with U_iso_(H) set to 1.2-1.5 *U*_eq_(C). The hydroxy and amino H-atoms were located in a difference Fourier map, and were refined isotropically with distance restraints of O–H, N–H = 0.85±0.01 Å.

### Crystal data

**Table:**

C_9_H_11_N_3_O_2_S	*Z* = 2
*M**_r_* = 225.27	*F*_000_ = 236
Triclinic, *P*1	*D*_x_ = 1.511 Mg m^−^^3^
Hall symbol: -P 1	Mo *K*α radiation λ = 0.71073 Å
*a* = 5.9932 (4) Å	Cell parameters from 558 reflections
*b* = 8.5207 (6) Å	θ = 2.9–23.0º
*c* = 10.3272 (6) Å	µ = 0.31 mm^−^^1^
α = 78.552 (4)º	*T* = 100 (2) K
β = 74.181 (4)º	Plate, yellow
γ = 81.743 (4)º	0.24 × 0.16 × 0.02 mm
*V* = 495.06 (6) Å^3^	

### Data collection

**Table:**

Bruker SMART APEX diffractometer	2258 independent reflections
Radiation source: fine-focus sealed tube	1580 reflections with *I* > 2σ(*I*)
Monochromator: graphite	*R*_int_ = 0.050
*T* = 100(2) K	θ_max_ = 27.5º
ω scans	θ_min_ = 2.5º
Absorption correction: Multi-scan(SADABS; Sheldrick, 1996)	*h* = −7→7
*T*_min_ = 0.929, *T*_max_ = 0.994	*k* = −11→8
4189 measured reflections	*l* = −13→13

### Refinement

**Table:**

Refinement on *F*^2^	Secondary atom site location: difference Fourier map
Least-squares matrix: full	Hydrogen site location: inferred from neighbouring sites
*R*[*F*^2^ > 2σ(*F*^2^)] = 0.054	H atoms treated by a mixture of independent and constrained refinement
*wR*(*F*^2^) = 0.123	*w* = 1/[σ^2^(*F*_o_^2^) + (0.0471*P*)^2^] where *P* = (*F*_o_^2^ + 2*F*_c_^2^)/3
*S* = 1.03	(Δ/σ)_max_ = 0.001
2258 reflections	Δρ_max_ = 0.32 e Å^−^^3^
153 parameters	Δρ_min_ = −0.47 e Å^−^^3^
4 restraints	Extinction correction: none
Primary atom site location: structure-invariant direct methods	

### Fractional atomic coordinates and isotropic or equivalent isotropic displacement parameters (Å^2^)

**Table:**

	*x*	*y*	*z*	*U*_iso_*/*U*_eq_	
S1	0.44298 (12)	0.47335 (9)	0.72966 (7)	0.0189 (2)	
O1	1.3132 (3)	0.8655 (2)	0.47568 (18)	0.0188 (4)	
O2	1.2446 (3)	1.1893 (2)	−0.03406 (18)	0.0200 (5)	
N1	0.8637 (4)	0.5405 (3)	0.7312 (2)	0.0147 (5)	
N2	0.7415 (4)	0.6387 (3)	0.5359 (2)	0.0159 (5)	
N3	0.9289 (4)	0.7298 (3)	0.4833 (2)	0.0145 (5)	
C1	0.8391 (5)	0.4576 (3)	0.8709 (2)	0.0188 (6)	
H1A	0.9802	0.4633	0.8996	0.028*	
H1B	0.8160	0.3447	0.8763	0.028*	
H1C	0.7044	0.5088	0.9310	0.028*	
C2	0.7001 (4)	0.5552 (3)	0.6642 (2)	0.0134 (6)	
C3	0.9368 (4)	0.8121 (3)	0.3637 (2)	0.0136 (6)	
H3	0.8213	0.8018	0.3194	0.016*	
C4	1.1147 (4)	0.9200 (3)	0.2937 (2)	0.0133 (6)	
C5	1.2915 (4)	0.9450 (3)	0.3503 (2)	0.0140 (6)	
C6	1.4505 (5)	1.0551 (3)	0.2796 (2)	0.0157 (6)	
H6	1.5673	1.0745	0.3193	0.019*	
C7	1.4389 (5)	1.1372 (3)	0.1504 (3)	0.0153 (6)	
H7	1.5496	1.2111	0.1016	0.018*	
C8	1.2669 (4)	1.1117 (3)	0.0929 (2)	0.0149 (6)	
C9	1.1055 (4)	1.0049 (3)	0.1638 (2)	0.0146 (6)	
H9	0.9866	0.9887	0.1243	0.018*	
H1o	1.206 (4)	0.805 (3)	0.511 (3)	0.048 (12)*	
H2o	1.352 (5)	1.245 (4)	−0.082 (3)	0.059 (13)*	
H1n	0.988 (3)	0.581 (4)	0.689 (3)	0.032 (9)*	
H2n	0.640 (4)	0.656 (4)	0.491 (3)	0.042 (10)*	

### Atomic displacement parameters (Å^2^)

**Table:**

	*U*^11^	*U*^22^	*U*^33^	*U*^12^	*U*^13^	*U*^23^
S1	0.0149 (4)	0.0241 (4)	0.0175 (3)	−0.0083 (3)	−0.0051 (3)	0.0033 (3)
O1	0.0222 (11)	0.0221 (12)	0.0140 (9)	−0.0081 (9)	−0.0089 (8)	0.0025 (8)
O2	0.0244 (11)	0.0202 (12)	0.0149 (9)	−0.0088 (9)	−0.0059 (8)	0.0047 (8)
N1	0.0114 (12)	0.0181 (13)	0.0148 (11)	−0.0035 (10)	−0.0038 (9)	−0.0007 (9)
N2	0.0153 (12)	0.0168 (13)	0.0158 (11)	−0.0060 (10)	−0.0056 (9)	0.0024 (9)
N3	0.0121 (11)	0.0136 (12)	0.0175 (11)	−0.0050 (9)	−0.0013 (9)	−0.0023 (9)
C1	0.0198 (15)	0.0231 (16)	0.0128 (12)	−0.0048 (12)	−0.0051 (11)	0.0015 (11)
C2	0.0137 (13)	0.0106 (14)	0.0155 (12)	−0.0011 (11)	−0.0032 (10)	−0.0022 (11)
C3	0.0136 (13)	0.0138 (14)	0.0147 (12)	−0.0019 (11)	−0.0053 (10)	−0.0025 (11)
C4	0.0134 (13)	0.0118 (14)	0.0153 (12)	−0.0008 (10)	−0.0034 (10)	−0.0038 (11)
C5	0.0137 (13)	0.0154 (15)	0.0133 (12)	0.0001 (11)	−0.0041 (10)	−0.0034 (11)
C6	0.0139 (13)	0.0162 (15)	0.0193 (13)	−0.0029 (11)	−0.0070 (10)	−0.0039 (11)
C7	0.0163 (14)	0.0102 (14)	0.0178 (13)	−0.0021 (11)	−0.0024 (10)	−0.0006 (11)
C8	0.0160 (14)	0.0144 (15)	0.0143 (12)	0.0006 (11)	−0.0050 (10)	−0.0023 (11)
C9	0.0158 (14)	0.0156 (15)	0.0140 (12)	−0.0021 (11)	−0.0059 (10)	−0.0031 (11)

### Geometric parameters (Å, °)

**Table:**

S1—C2	1.695 (3)	C1—H1B	0.9800
O1—C5	1.367 (3)	C1—H1C	0.9800
O1—H1o	0.84 (3)	C3—C4	1.450 (4)
O2—C8	1.379 (3)	C3—H3	0.9500
O2—H2o	0.84 (3)	C4—C5	1.401 (4)
N1—C2	1.325 (3)	C4—C9	1.403 (3)
N1—C1	1.453 (3)	C5—C6	1.388 (4)
N1—H1n	0.84 (3)	C6—C7	1.393 (3)
N2—C2	1.349 (3)	C6—H6	0.9500
N2—N3	1.382 (3)	C7—C8	1.383 (4)
N2—H2n	0.84 (3)	C7—H7	0.9500
N3—C3	1.286 (3)	C8—C9	1.379 (4)
C1—H1A	0.9800	C9—H9	0.9500
			
C5—O1—H1O	110 (2)	C4—C3—H3	118.6
C8—O2—H2O	117 (3)	C5—C4—C9	118.9 (2)
C2—N1—C1	123.8 (2)	C5—C4—C3	123.2 (2)
C2—N1—H1N	117 (2)	C9—C4—C3	117.9 (2)
C1—N1—H1N	119 (2)	O1—C5—C6	117.7 (2)
C2—N2—N3	121.3 (2)	O1—C5—C4	122.3 (2)
C2—N2—H2N	122 (2)	C6—C5—C4	120.0 (2)
N3—N2—H2N	116 (2)	C5—C6—C7	120.1 (3)
C3—N3—N2	114.5 (2)	C5—C6—H6	120.0
N1—C1—H1A	109.5	C7—C6—H6	120.0
N1—C1—H1B	109.5	C8—C7—C6	120.3 (2)
H1A—C1—H1B	109.5	C8—C7—H7	119.9
N1—C1—H1C	109.5	C6—C7—H7	119.9
H1A—C1—H1C	109.5	O2—C8—C9	116.7 (2)
H1B—C1—H1C	109.5	O2—C8—C7	123.3 (2)
N1—C2—N2	118.2 (2)	C9—C8—C7	119.9 (2)
N1—C2—S1	123.80 (19)	C8—C9—C4	120.8 (2)
N2—C2—S1	118.0 (2)	C8—C9—H9	119.6
N3—C3—C4	122.7 (2)	C4—C9—H9	119.6
N3—C3—H3	118.6		
			
C2—N2—N3—C3	−175.1 (2)	C3—C4—C5—C6	−177.4 (2)
C1—N1—C2—N2	178.3 (2)	O1—C5—C6—C7	178.8 (2)
C1—N1—C2—S1	−2.5 (4)	C4—C5—C6—C7	−2.0 (4)
N3—N2—C2—N1	−11.0 (4)	C5—C6—C7—C8	1.1 (4)
N3—N2—C2—S1	169.69 (19)	C6—C7—C8—O2	179.7 (2)
N2—N3—C3—C4	177.5 (2)	C6—C7—C8—C9	0.2 (4)
N3—C3—C4—C5	−0.6 (4)	O2—C8—C9—C4	179.8 (2)
N3—C3—C4—C9	−179.5 (2)	C7—C8—C9—C4	−0.7 (4)
C9—C4—C5—O1	−179.3 (2)	C5—C4—C9—C8	−0.2 (4)
C3—C4—C5—O1	1.8 (4)	C3—C4—C9—C8	178.8 (2)
C9—C4—C5—C6	1.5 (4)		

### Hydrogen-bond geometry (Å, °)

**Table:**

*D*—H···*A*	*D*—H	H···*A*	*D*···*A*	*D*—H···*A*
O1—H1o···N3	0.84 (3)	1.97 (2)	2.698 (3)	144 (3)
O2—H2o···S1^i^	0.84 (3)	2.46 (2)	3.182 (2)	144 (3)
N2—H2n···O1^ii^	0.84 (3)	2.47 (3)	3.111 (3)	134 (3)

Symmetry codes: (i) *x*+1, *y*+1, *z*−1; (ii) *x*−1, *y*, *z*.

## References

[bb1] Barbour, L. J. (2001). *J. Supramol. Chem.* **1**, 189–191.

[bb2] Bruker (2007). *APEX2* and *SAINT* Bruker AXS Inc., Madison, Wisconsin, USA.

[bb3] Kayed, S. F., Farina, Y., Baba, I. & Simpson, J. (2008). *Acta Cryst.* E**64**, o824–o825. 10.1107/S1600536808009148 PMC2961087 21202314

[bb4] Libermann, D., Moyeux, M., Rouaix, A., Maillard, J., Hengl, L. & Himbert, J. (1953). *Bull. Soc. Chim. Fr.* pp. 957–962.

[bb5] Sheldrick, G. M. (1996). *SADABS* University of Göttingen, Germany.

[bb6] Sheldrick, G. M. (2008). *Acta Cryst.* A**64**, 112–122. 10.1107/S0108767307043930 18156677

[bb7] Taniyama, H. & Tanaka, Y. (1965). *Yakugaku Kenkyu*, **36**, 319–328.

[bb8] Vrdoljak, V., Cindrić, M., Milić, D., Matković-Čalogović, D., Novak, P. & Kamenar, B. (2005). *Polyhedron*, **24**, 1717–1726.

[bb9] Westrip, S. P. (2008). *publCIF* In preparation.

[bb10] Xue, C.-B., Zhang, L., Luo, W.-C., Xie, X.-Y., Jiang, L. & Xiao, T. (2007). *Bioorg. Med. Chem.* **15**, 2006–2015. 10.1016/j.bmc.2006.12.038 17258462

